# Centrifugal progression of retinal lesions in the early evolution of multiple evanescent white dot syndrome

**DOI:** 10.1007/s00417-023-06226-7

**Published:** 2023-09-08

**Authors:** Ariel Yuhan Ong, Katrina Fordwor, Peter Charbel Issa

**Affiliations:** 1 Oxford Eye Hospital, Oxford University Hospitals NHS Foundation Trust, John Radcliffe Hospital, Oxford, OX3 9DU UK; 2 Nuffield Laboratory of Ophthalmology, Nuffield Department of Clinical Neurosciences, University of Oxford, Oxford, UK; 3 Oxford University Hospitals NHS Foundation Trust, Oxford, UK

**Keywords:** Multiple evanescent white dot syndrome, MEWDS, Autofluorescence


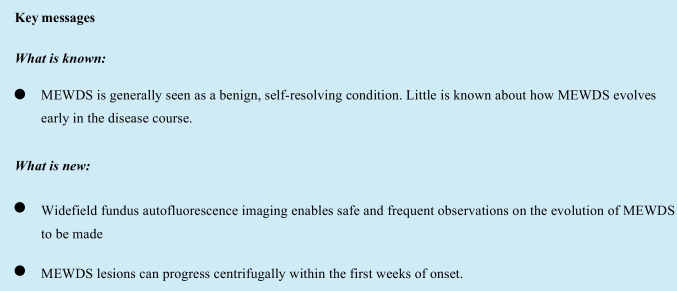


Multimodal imaging is often essential in confirming the diagnosis of multiple evanescent white dot syndrome (MEWDS). Fundus autofluorescence (FAF) imaging appears to be particularly sensitive in detecting MEWDS lesions and may best be used to illustrate the extent and topographic distribution of MEWDS lesions [1]. Non-invasive ultra-widefield FAF imaging enables safe and quick repeat imaging, often does not require pupil dilation, and avoids the potential risks associated with angiography. Little is known about possible short-term changes of MEWDS early in the disease course. Here, we show dynamic changes in the topographic distribution of MEWDS lesions within the first weeks after symptom onset illustrated with FAF.

**Fig. 1:**
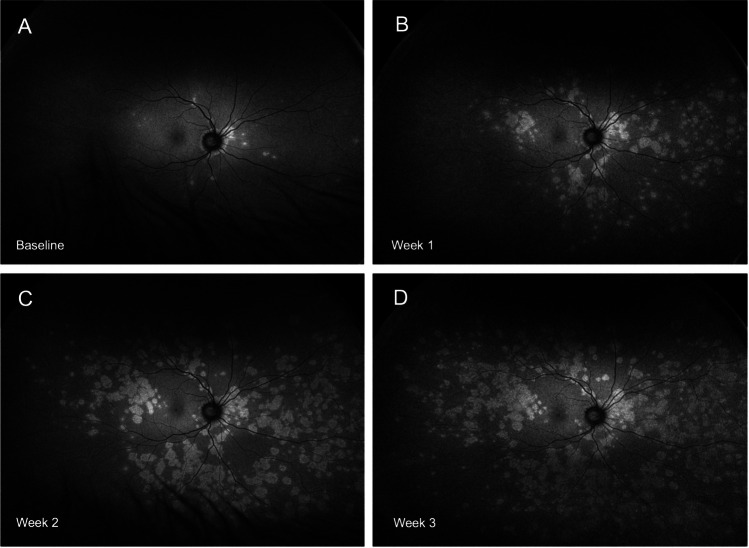
Widefield FAF imaging (patient #1) demonstrating centrifugal propagation of MEWDS lesions: at baseline (the initial visit 2 days after symptom onset) (**A**), week 1 with worsening photopsia (**B**), week 2 with increasing nasal and temporal scotomata (**C**), and week 3 with stable symptoms (**D**)

Patient 1 was a 33-year-old female who presented with a 2-day history of a paracentral scotoma and photopsias in her right eye. Visual acuity was −0.1 logMAR bilaterally. She reported worsening symptoms over the next 2 weeks. Widefield FAF imaging demonstrated centrifugal spread of the MEWDS lesions, which were initially circumpapillary and progressed to encompass the peripheral retina with sparing of the fovea (Fig. 1).

**Fig. 2:**
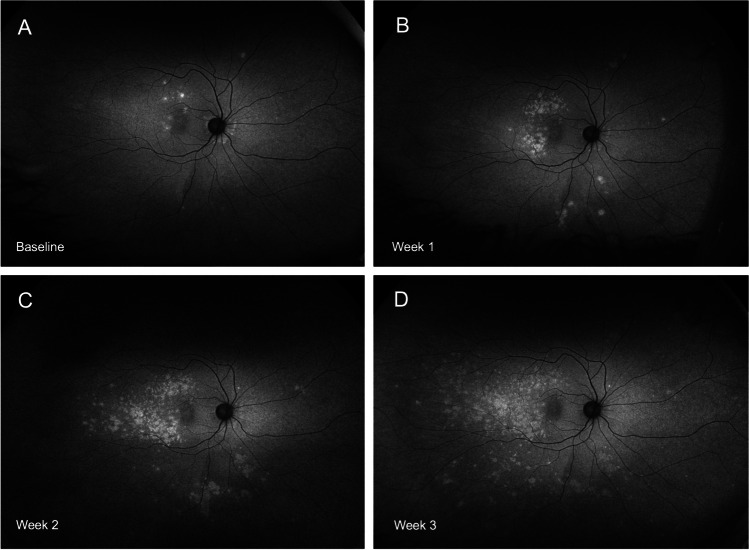
Widefield FAF imaging (patient #2) demonstrating centrifugal propagation of MEWDS lesions predominantly affecting the temporal and inferior retina: at baseline (the initial visit 2 days after symptom onset) (**A**), week 1 with worsening symptoms (**B**), week 2 with an improvement in symptoms (**C**), and week 3 with stable symptoms (**D**)

Patient 2 was a 45-year-old male who presented with a 2-day history of central and paracentral scotomata in his right eye. Visual acuity was 0.2 and −0.2 logMAR in the affected and fellow eye, respectively. Initial FAF imaging showed MEWDS lesions mainly in the juxtapapillary and macular area. Over the following 2–3 weeks, additional MEWDS lesions developed at increasing eccentricity with relative fovea sparing (Fig. 2).

Both patients had moderate myopia and characteristic features of MEWDS on multimodal imaging, which included color fundus photography, fluorescein and ICG angiography, and OCT imaging (not shown). In both cases, the MEWDS lesions started in the peripapillary region and subsequently became more widespread over the next 2–3 weeks, progressing in a centrifugal pattern. These changes corresponded with the patients’ reports of progressive symptoms. Symptoms and retinal changes improved as expected in the ensuing weeks and follow-up intervals were increased. Symptoms and retinal changes on multimodal imaging had resolved 10 weeks (patient 1) and 5 months (patient 2) after initial presentation. Neither had any antecedent viral symptoms.

We found 4 cases in the literature that demonstrated similar findings of initial circumpapillary changes with subsequent progression, albeit without the benefit of widefield imaging [2–5]. Similar to these cases, our patients presented within a few days of symptom onset and were monitored at short intervals, but additionally using serial ultra-widefield FAF imaging. Possible explanations for why early dynamic changes of MEWDS lesions are not commonly documented may be that 1) patients are often seen with longer delay after symptom onset and followed up many weeks later because of the anticipated spontaneous improvement, meaning that the initial short-term changes of MEWDS lesions are not captured; 2) some MEWDS events might remain limited (e.g., to the peripapillary area), while others progress to retina-wide involvement; or 3) evolution dynamics of the lesions might differ between cases, i.e., MEWDS lesions may already have fully developed at first presentation of patients with much faster progression than in the cases presented here.

It remains to be determined whether the early evolution of MEWDS lesions represents an actual centrifugal spread or if the triggering event occurs at all affected locations simultaneously, but with differing rates of developing visible lesions depending on eccentricity. Moreover, whether a similar centrifugal propagation of MEWDS lesion occurs in cases where MEWDS lesions are secondary to and centered on a pre-existing chorioretinal lesion remains to be established.

In summary, we show substantial progression of MEWDS lesions within the first weeks after symptom onset. Such dynamic changes need to be taken into account when determining severity and topographic distribution of MEWDS lesions, as conclusions may depend on the actual time point of the assessment.
